# Multidrug-Resistant Methicillin-Resistant *Staphylococcus aureus* Associated with Bacteremia and Monocyte Evasion, Rio de Janeiro, Brazil

**DOI:** 10.3201/eid2711.210097

**Published:** 2021-11

**Authors:** Alice Slotfeldt Viana, Ana Maria Nunes Botelho, Ahmed M. Moustafa, Craig L.K. Boge, Adriana Lucia Pires Ferreira, Maria Cícera da Silva Carvalho, Márcia Aparecida Guimarães, Bruno de Souza Scramignon Costa, Marcos Corrêa de Mattos, Sabrina Pires Maciel, Juliana Echevarria-Lima, Apurva Narechania, Kelsey O’Brien, Chanelle Ryan, Jeffrey S. Gerber, Bernadete Teixeira Ferreira Carvalho, Agnes Marie Sá Figueiredo, Paul J. Planet

**Affiliations:** Universidade Federal do Rio de Janeiro, Rio de Janeiro, Brazil (A.S. Viana, A.M.N. Botelho, A.L.P. Ferreira, M.C.S. Carvalho, M.A. Guimarães, B.S.S. Costa, M.C. Mattos, S.P. Maciel, J. Echevarria-Lima, B.T.F. Carvalho, A.M.S. Figueiredo);; Children’s Hospital of Philadelphia, Philadelphia, Pennsylvania, USA (A.M. Moustafa, C.L.K. Boge, K. O’Brien, C. Ryan, J.S. Gerber, P.J. Planet);; Diagnósticos da América S.A., Duque de Caxias, Brazil (A.L.P. Ferreira);; American Museum of Natural History, New York, New York, USA (A. Narechania, P.J. Planet);; University of Pennsylvania, Philadelphia (J.S. Gerber, P.J. Planet)

**Keywords:** MRSA, molecular epidemiology, phylogenetics, bloodstream infections, monocytes, phagocytosis, methicillin-resistant *Staphylococcus aureus*, *Staphylococcus aureus*, drug resistance, antimicrobial resistance, multidrug-resistance, bacteremia, monocyte evasion, bacteria, bacterial infection, Rio de Janeiro, Brazil, MRSA and other staphylococci

## Abstract

We typed 600 methicillin-resistant *Staphylococcus aureus* (MRSA) isolates collected in 51 hospitals in the Rio de Janeiro, Brazil, metropolitan area during 2014–2017. We found that multiple new clonal complex (CC) 5 sequence types had replaced previously dominant MRSA lineages in hospitals. Whole-genome analysis of 208 isolates revealed an emerging sublineage of multidrug-resistant MRSA, sequence type 105, staphylococcal cassette chromosome *mec* II, *spa* t002, which we designated the Rio de Janeiro (RdJ) clone. Using molecular clock analysis, we hypothesized that this lineage began to expand in the Rio de Janeiro metropolitan area in 2009. Multivariate analysis supported an association between bloodstream infections and the CC5 lineage that includes the RdJ clone. Compared with other closely related isolates, representative isolates of the RdJ clone more effectively evaded immune function related to monocytic cells, as evidenced by decreased phagocytosis rate and increased numbers of viable unphagocytosed (free) bacteria after in vitro exposure to monocytes.

Methicillin-resistant *Staphylococcus aureus* (MRSA) is characterized by the mainly clonal structure of bacterial populations and the worldwide spread of a few highly successful lineages, sequence types (STs), and clonal complexes (CCs) that cycle through waves of dominance (*1*,*2*). During the late 1990s, the Brazilian endemic clone (BEC), which belongs to the ST239(CC8)–staphylococcal cassette chromosome (SCC) *mec*III lineage, comprised ≈80% of MRSA isolates in hospitals in Brazil (*3*). In the 2000s, isolates of the ST1(CC1)-SCC*mec*IV lineage supplanted BEC in >2 hospitals in the Rio de Janeiro metropolitan area of Brazil (*4*). More recent analyses have suggested that CC5 isolates might be increasing in prevalence in Brazil (*5*).

Most studies on the molecular epidemiology of MRSA in Brazil have analyzed a small number of isolates from a limited number of hospitals (*5*–*9*). We used molecular and genomic approaches to characterize 600 MRSA isolates collected from 51 hospitals in the Rio de Janeiro metropolitan area and identified a novel MRSA clone of ST105-SCC*mec*II *spa* t002 (ST105-SCC*mec*II-t002), which we termed the Rio de Janeiro (RdJ) clone, as a predominant cause of MRSA bloodstream infections (BSIs).

## Methods

### Bacterial Isolates

We obtained the MRSA isolates from 600 patients at 51 hospitals in the Rio de Janeiro metropolitan area and confirmed MRSA using routine identification methods (Table 1; Appendix 1). The sample comprised roughly equal numbers of isolates from blood samples from BSI patients, nonblood samples from patients with infections at another body site, and nasal swab samples; samples were collected during 2014–2017, most in 2015 and 2016. Patient age was available for 450 patients (Table 2). The research protocols were submitted to the Human Research Ethics Committee (CAAE submission no. 41614914.4.00005257) of the Hospital Universitário Clementino Fraga Filho, Universidade Federal do Rio de Janeiro (Rio de Janeiro, Brazil); the study was considered non–human subject research.

**Table 1:** Sample types of methicillin-resistant *Staphylococcus aureus* isolates from colonized and infected patients, Rio de Janeiro, Brazil, 2014–2017

Sample type	No. (%) samples
Blood	197 (32.8)
Nonblood	216 (36.0)
Anterior nasal swab	187 (31.2)
Total	600 (100.0)

**Table 2:** Age distribution of patients who had methicillin-resistant *Staphylococcus aureus* infections or colonizations, Rio de Janeiro, Brazil, 2014–2017

Patient age range, y	No. (%)
<5	46 (10.2)
5–18	16 (3.6)
19–59	180 (40.0)
>60	208 (46.2)
Total	450 (100.0)

### Molecular Typing and Susceptibility Testing

We used restriction-modification (RM) tests to determine CC (*10*) and multiplex PCR to type SCC*mec* (*11*). We used PCR to screen for the *lukSF-PV*, *agr*II, SCC*mec*III, and *seh* genes as previously described (*12*). We conducted antibiogram and susceptibility tests for glycopeptide drugs as recommended by Clinical and Laboratory Standards Institute guidelines (*13*).

### Genome Sequencing and Analysis

We selected 208 isolates for whole-genome sequencing (WGS). Because of a strong predominance (179/208; 86.1%) of CC5 isolates, we focused our research on the CC5 lineage. We randomly selected isolates from blood (70/145; 48.3%), nonblood (52/114; 45.6%), and nasal swab (57/123; 46.3%) CC5 samples (Appendix 2). The other 29 isolates used in WGS belonged to less abundant CCs. We prepared genomic DNA using the Wizard Genomic DNA Purification Kit (Promega Corporation, https://www.promega.com) and sequenced genome libraries by using Nextera XT DNA Library Prep Kit (Illumina, https://www.illumina.com) and the HiSeq 2500 system (Illumina) using paired-end reads of 125 bp. We trimmed reads using BBDuk Trimmer version 1.0 (Geneious, https://www.geneious.com) and assembled genomes using Velvet Assembly version 7.0.4 (*14*) and SPAdes version 3.13.0 (*15*). We used RAST (https://rast.nmpdr.org) and manual inspection to annotate the isolates. We determined the genotypes of the sequenced strains using the MLST 2.0, SCC*mec*Finder 1.2, and *spa* Typer 1.0 tools (https://cge.cbs.dtu.dk).

### Phylogenetic Analysis and Divergence Times

We constructed a maximum-likelihood tree for 661 CC5 genomes: 179 genomes from the current investigation and 482 assembled genomes available on GenBank, chosen from the list provided by Challagundla et al. (*8*) (Appendix 3). We used a single-nucleotide polymorphism (SNP) alignment produced by Snippy to infer an initial phylogenetic tree in RAxML version 8.2.4 (*16*).

To estimate when the ST105-SCC*mec*II-t002 lineage emerged in Rio de Janeiro, we used a Bayesian phylogenetic framework to analyze 73 genomes that passed our Mash Screen (*17*) quality cutoffs. We selected MRSA strain FCFHV36, the closest complete reference genome available in GenBank, using the WhatsGNU topgenome (-t) option (*18*). We used the SNP alignment to infer an initial phylogenetic tree in RAxML version 8.2.4 before using ClonalFrameML (*19*) to detect and mask areas of recombination. We used the SNP recombination-masked alignment to estimate divergence times in BEAST version 2.6.2 (*20*). We found a positive correlation between genetic divergence and isolation time using TempEst version 1.5.3 (*21*). We plotted the chronograms based on the maximum clade credibility tree using the TreeAnnotator program and visualized in FigTree version 1.4.3 (Appendix 3).

### Genomic Island Characterization

**Figure 1:**
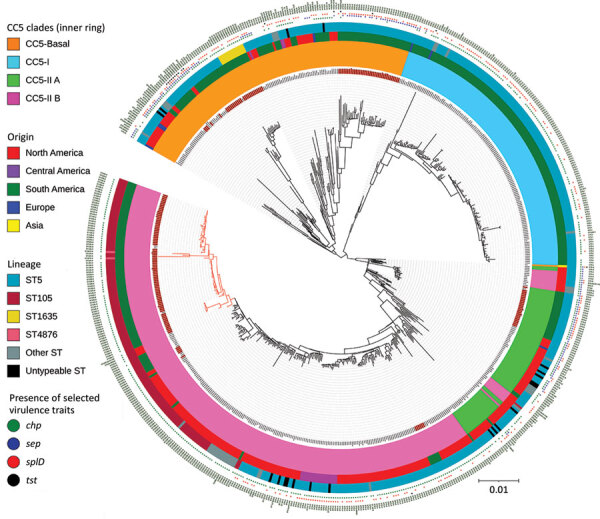
Maximum-likelihood phylogenetic tree of 179 methicillin-resistant *Staphylococcus aureus* CC5 isolates from Rio de Janeiro, Brazil, 2014–2017 (red text) and 482 reference genomes (*7*). Red branches indicate the Rio de Janeiro clone of the lineage ST105(CC5)-SCC*mec*II-t002. Scale indicates substitutions per site. CC, clonal complex; SCC, staphylococcal chromosome cassette; ST, sequence type.

We used Geneious Prime version 2020.1.2 to manually inspect the ΦSA3, vSa-α, vSa-β, vSa-ɣ, and SaPI-1 genomic islands (*22*,*23*) and Swiss-Prot (Uniprot Consortium, https://www.uniprot.org) to annotate paralogues. To map the genetic context of genomic islands, we randomly selected representative genome sequences from different phylogenetic locations of the tree showing the most common CC5 lineages in the Rio de Janeiro metropolitan area (Figure 1). We determined gene presence or absence using BLAST analysis (https://blast.ncbi.nlm.nih.gov).

### Phagocytosis Assays

We subjected the selected isolates to phagocytosis (Appendix 3 Table 1). In this assay, we considered the entire process of phagocytosis (i.e., binding and uptake) by detecting all cell-associated bacteria, whether internalized or externally attached, after washing. We cultured bacteria at 37°C for 18 h at 250 rpm in brain–heart infusion broth (Becton Dickinson, https://www.bd.com) before treating with 25 nmol SYTO 9 stain (Thermo Fisher Scientific, https://www.thermofisher.com) for 15 min and washing in phosphate-buffered saline (1× phosphate-buffered saline, pH 7.2). We incubated bacterial cells at 37°C for 30 min in 5% carbon dioxide with THP-1 monocytes in Roswell Park Memorial Institute 1640 medium for a multiplicity of infection of 10 (*24*). We did not use antimicrobial drugs at any time during these assays. We washed the infected monocytes with PBS once and then centrifuged them at 200 × *g* for 5 min. We resuspended THP-1 cells in PBS and analyzed them by flow cytometry (FACSCalibur; Becton Dickinson). We acquired 10,000 live THP-1 cells (as calculated by forward scatter and side scatter gating) and analyzed data using FlowJo10 software (https://www.flowjo.com). We calculated the number of bacteria-associated THP-1 cells as the frequency of fluorescent (i.e., SYTO 9–positive) THP-1 cells compared with total live THP-1 cells. In addition, we counted and compared the number of viable unphagocytosed bacterial cells in the culture supernatant of each assay at 0 and 30 min after incubation.

### Statistical Analyses

We analyzed molecular typing, antimicrobial testing, and epidemiologic data using Pearson χ^2^ tests. To assess the association of the CC5-SCC*mec*II group and the ST105-SCC*mec*II-t002 sublineage with BSI, we used Stata 16.0 (https://www.stata.com) to conduct a Mantel-Haenszel test stratified on a composite variable informed by participant age (>60 years vs. <60 years), year of specimen collection (2014, 2015, or 2016–2017), and hospital type (public vs. private). For the analysis of year of specimen collection, we combined data from 2016 and 2017 because few isolates were collected during 2017. We analyzed phagocytosis assays using a 1-way analysis of variance and Tukey multiple comparison test in GraphPad Prism 6 (GraphPad Software, Inc., https://www.graphpad.com).

## Results

### Distribution of Genotypes (CC-SCC*mec*) and Antimicrobial Resistance

**Figure 2:**
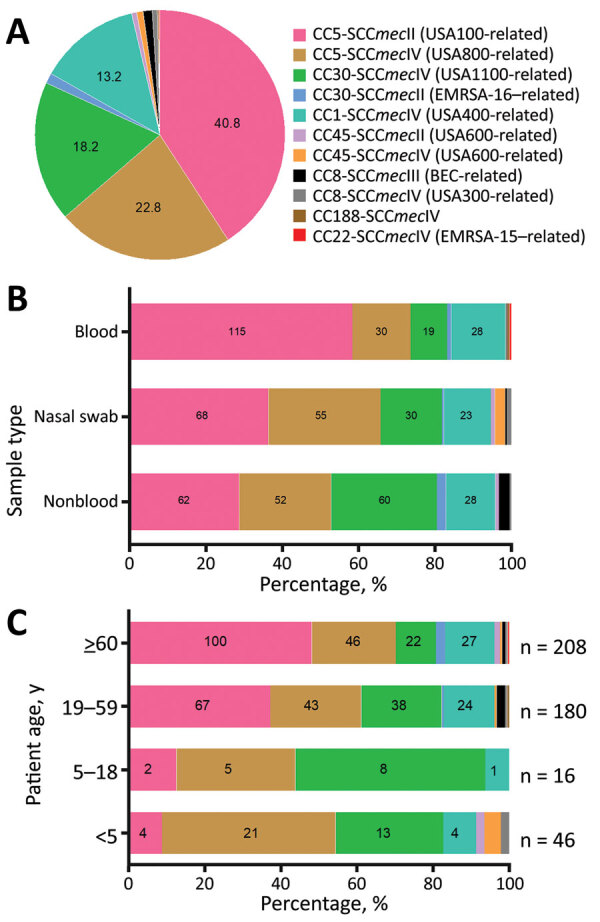
Distribution of 600 MRSA isolates by lineage (A), sample type (B), and patient age (C), Rio de Janeiro Brazil, 2014–2017. A) MRSA isolates by lineage (CC-SCC*mec* type) among 600 isolates. Labels indicate proportions. B) MRSA isolates by sample type. Labels indicate number of isolates. C) MRSA isolates by patient age (data available for 450 patients). Labels indicate number of isolates. BEC, Brazilian endemic clone; CC, clonal complex; EMRSA, epidemic methicillin-resistant *Staphylococcus aureus*; MRSA, methicillin-resistant *Staphylococcus aureus*; SCC, staphylococcal cassette chromosome.

Among the 600 isolates that underwent CC and SCC*mec* typing, most were categorized as CC5-SCC*mec*II (245/600; 40.8%) or CC5-SCC*mec*IV (137/600; 22.8%). The second most common lineage was CC30, comprised of *lukSF-PV*–positive CC30-SCC*mec*IV (109/600; 18.2%) and *lukSF-PV*–negative CC30-SCC*mec*II (8/600; 1.3%) isolates. The previously dominant CC1-SCC*mec*IV lineage (79/600; 13.2%) and BEC clone (7/600; 1.2%) were much less frequent. In addition, we observed low frequencies of STs related to other international lineages such as CC45-SCC*mec*II/IV (related to USA600), CC8-SCC*mec*IV (related to USA300), and CC22-SCC*mec*IV (related to EMRSA-15) (Figure 2, panel A).

Compared with isolates of other frequent clonal lineages, CC5-SCC*mec*II isolates were more likely to be multidrug-resistant, defined as having resistance to >4 non-β-lactam antimicrobial drugs (48.6% vs. 5.8%) (Table 3). In contrast, CC5-SCC*mec*IV strains showed more susceptibility to non–β-lactams; only 4.4% were multidrug-resistant. All 109 strains belonging to the CC30-SCC*mec*IV lineage, which is related to the community-acquired MRSA USA1100/Oceania South West Pacific clone, were susceptible to all non–β-lactams tested (Table 3).

**Table 3:** Antimicrobial resistance among 600 methicillin-resistant *Staphylococcus aureus* isolates, Rio de Janeiro, Brazil, 2014–2017*

Lineage	Total	No. multidrug-resistant isolates, %
CC5-SCC*mec*II	245	119 (48.6)†
CC5-SCC*mec*IV	137	6 (4.4)
CC30-SCC*mec*IV	109	0
CC1-SCC*mec*IV	79	13 (16.5)
CC30-SCC*mec*II	8	3 (37.5)
CC8-SCC*mec*III	7	7 (100.0)
CC45-SCC*mec*IV	5	0
CC45-SCC*mec*II	4	2 (50.0)
CC8-SCC*mec*IV	4	0
CC188-SCC*mec*IV	1	0
CC22-SCC*mec*IV	1	0

*Multidrug-resistant defined as an isolate carrying >4 additional antimicrobial resistance traits to non–β-lactam antimicrobial drugs. †p<0.01 by Pearson χ^2^ test.

###  Distribution of Genotypes (CC-SCC*mec*) and Clinical Data

In the univariate analysis, we found that the distribution of genotypes was associated with MRSA infection site (Figure 2, panel B). CC5-SCC*mec*II isolates were more common among blood (115/245; 46.9%) than nonblood (62/245; 25.3%) and nasal swab (68/245; 27.8%) samples, whereas CC5-SCC*mec*IV isolates were more common among nasal swab (55/137; 40.1%) and nonblood (52/137; 38.0%) than blood (30/137; 21.9%) samples. The third most frequent lineage, CC30-SCC*mec*IV, was more common among nonblood (60/109; 55.0%) than nasal swab (30/109; 27.5%) and blood (19/109; 17.4%) samples.

The distribution of MRSA lineages varied among age groups. CC5-SCC*mec*II was more common among patients >60 years of age (100/208; 48.1%). CC30-SCC*mec*IV prevalence was higher among younger populations and diminished with increasing age range; prevalence was 50.0% (8/16) among children 5–18 years of age, 21.1% (38/180) among adults 19–59 years of age, and 10.6% (22/208) among adults >60 years of age. Among children <5 years of age, the most prevalent lineage was CC5-SCC*mec*IV, which is sometimes known as the pediatric clone (21/46; 45.7%) (Figure 2, panel C). Adults 19–59 and >60 years of age had a similar prevalence of CC5-SCC*mec*IV isolates (23.9% among adults 19–59 years of age vs. 22.1% among adults >60 years of age). The proportion of CC5-SCC*mec*II isolates was also similar between adults 19–59 years of age (57/180; 31.7%) and adults >60 years of age (62/208; 29.8%). CC5-SCC*mec*II was associated with BSIs even after stratifying for the composite variable of age, hospital type, and year of isolation (p<0.01).

### Novel MRSA Clone

To better characterize the circulating clones, especially those belonging to CC5, we used whole-genome sequencing on 208 isolates: 76 (36.5%) from blood samples, 69 (33.2%) from nasal swab samples, and 63 (30.3%) from nonblood samples. Most (179; 86.1%) isolates belonged to CC5, whereas 29 did not (Appendix 3 Table 2). Multilocus and *spa*-typing using WGS revealed 4 CC5 clones that constituted >75% of isolates (Table 4). The dominant genotype, ST105(CC5)-SCC*mec*II-t002, the RdJ clone, comprised 41.9% (75/179) of the CC5 isolates. RdJ showed the second highest proportion of multidrug resistance (41/75; 54.7%), superseded only by ST5-SCC*mec*II-t539 (14/17; 82.4%). In contrast, only 1 (2.3%) strain of ST5-SCC*mec*IV-t002 was multidrug-resistant. MRSA lineages coexisting in the same hospital often displayed different resistance profiles.

**Table 4:** Lineages of methicillin-resistant *Staphylococcus aureus* clonal complex 5 isolates, Rio de Janeiro, Brazil, 2014–2017*

Clones†	Blood	Anterior nasal swab	Nonblood	Total (%)
ST105-SCC*mec*II-t002	41	20	14	75 (41.9)‡
ST5-SCC*mec*IV-t002	11	13	19	43 (24.0)
ST5-SCC*mec*II-t539	5	4	8	17 (9.5)
ST1635-SCC*mec*IV-t002	2	4	4	10 (5.6)
ST5-SCC*mec*II-t067	2	3	1	6 (3.4)
ST5-SCC*mec*II-t2666	2	2	1	5 (2.8)
ST105-SCC*mec*II-NT	3	0	0	3 (1.7)
ST105-SCC*mec*II-t010	1	0	1	2 (1.1)
ST4876-SCC*mec*II-t002	1	0	1	2 (1.1)
ST5-SCC*mec*IV-t1154	1	1	0	2 (1.1)
ST5-SCC*mec*IV-NT	0	2	0	2 (1.1)
ST105-SCC*mec*II-t067	1	0	0	1 (0.6)
ST105-SCC*mec*II-t539	0	1	0	1 (0.6)
ST1635-SCC*mec*IV-t062	0	0	1	1 (0.6)
ST1635-SCC*mec*IV-t450	0	1	0	1 (0.6)
ST1635-SCC*mec*IV-t769	0	0	1	1 (0.6)
ST5-SCC*mec*II-t002	0	1	0	1 (0.6)
ST5-SCC*mec*II-NT	0	1	0	1 (0.6)
ST5-SCC*mec*IV-t061	0	1	0	1 (0.6)
ST5-SCC*mec*IV-t062	0	0	1	1 (0.6)
ST5-SCC*mec*IV-t105	0	1	0	1 (0.6)
ST5-SCC*mec*IV-t586	0	1	0	1 (0.6)
ST5-SCC*mec*IV-t777	0	1	0	1 (0.6)
Total sequenced/collected§	70/145	57/123	52/114	179/382

*SCC, staphylococcal chromosome cassette; NT, not typed by *spa* polymorphism; ST, sequence type. †Clones defined by multilocus sequence type, SCC*mec* type, and *spa* polymorphism. ‡p<0.01 by single-variable analysis for blood samples compared with nasal swab and nonblood samples; p = 0.12 when adjusted for private vs. public hospital and year of isolation. §Total isolates sequenced from total no. isolates collected.

In addition to being the most frequent MRSA clone, RdJ might be responsible for the higher frequency of CC5-SCC*mec*II isolates from blood samples. ST105-SCC*mec*II-t002 isolates were more common among blood (41/75; 54.7%) than nasal swab (20/75; 26.7%) and nonblood (14/75; 18.7%) samples; however, when adjusted for hospital type and year of isolation, this association became nonsignificant (p = 0.12).

### Whole-Genome Phylogenetic Analysis of MRSA CC5 Isolates

**Figure 3:**
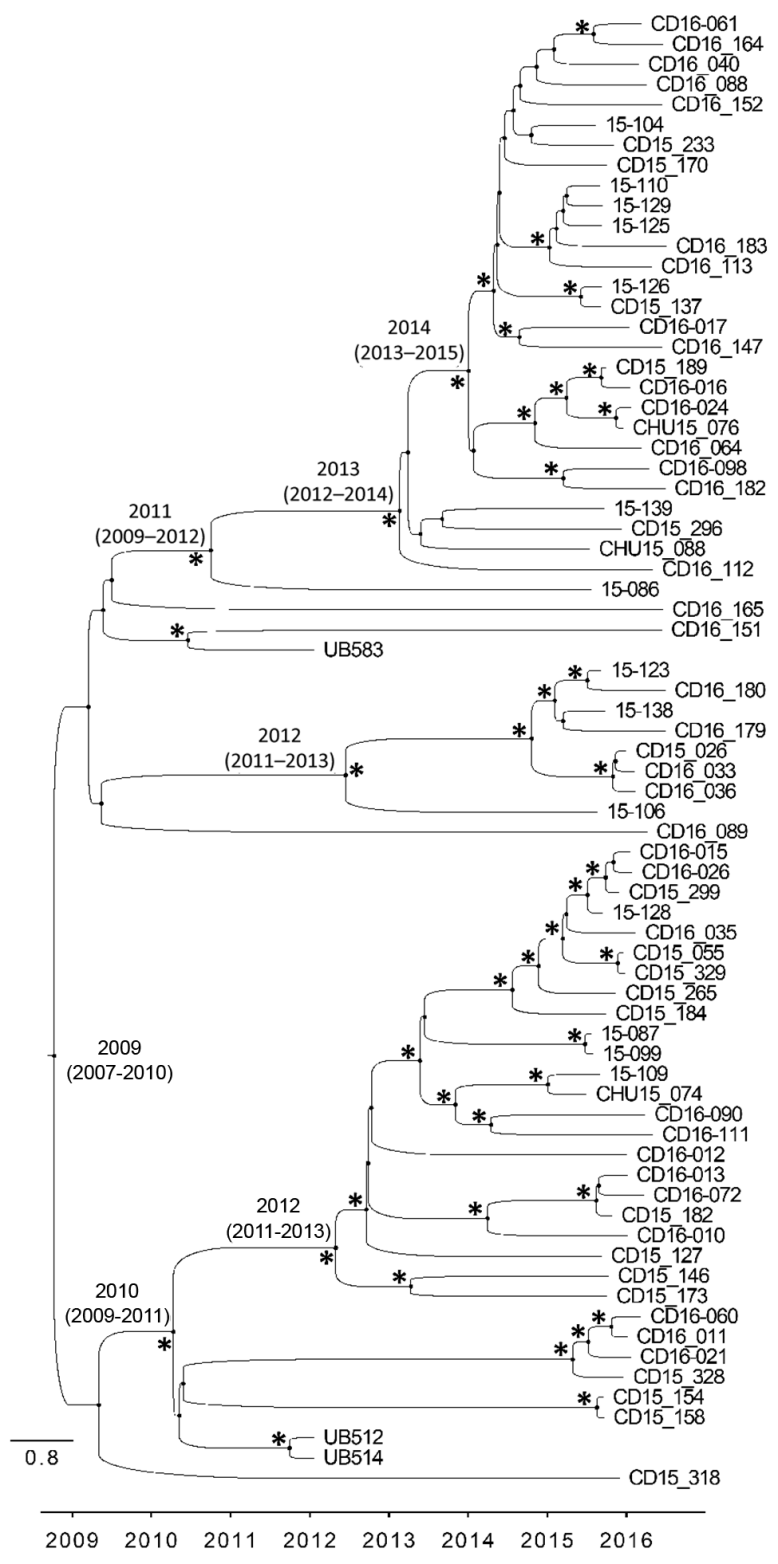
Time-calibrated phylogenetic tree of methicillin-resistant *Staphylococcus aureus* ST105(CC5)-SCC*mec*II-t002 lineage, Rio de Janeiro, Brazil, 2014–2017. Chronogram constructed using Bayesian phylogenetic analysis of single-nucleotide polymorphisms from 73 genomes. Maximum clade credibility tree estimated using a strict clock rate of 1.1927 × 10^–6^ substitutions/site/year (95% highest posterior density 1.5054–2.3351 × 10^–6^). Node labels indicate 95% highest posterior density values of major clades. Asterisks (*) indicate posterior values >0.98. Scale indicates substitutions per site per year. CC, clonal complex; SCC, staphylococcal cassette chromosome; ST, sequence type.

The whole-genome phylogenetic analysis grouped CC5 isolates from this study into 3 of the 4 major phylogenetic groups corresponding mostly to the ST105(CC5)-SCC*mec*II-t002, ST5-SCC*mec*II-t539, and ST5-SCC*mec*IV-t002 genotypes and distributed widely throughout the CC5 tree (Figure 1). All SCC*mec*IV isolates clustered in the CC5-Basal clade. Isolates with the multidrug-resistant ST5(CC5)-SCC*mec*II-t539 genotype clustered with members of the paraphyletic group CC5-IIA described by Challagundla et al. (*8*). Most other CC5 isolates, including isolates of genotype ST105(CC5)-SCC*mec*II-t002, were grouped in clade CC5-IIB. Although most of these isolates form the RdJ clade, which is found mostly in Rio de Janeiro, nearby outgroups to this clade are composed of previously sequenced isolates from São Paulo and Porto Alegre (*25*) and North America, as well as a few isolates from this study (Appendix 3). This pattern might indicate multiple introductions into Brazil. Our Bayesian analysis of the RdJ clade suggests a recent date of introduction, probably 2009 (95% highest posterior density 2007–2010) (Figure 3).

In comparison with other CC5 genomes, the clade that includes the ST105 genomes lacked key virulence genes. In addition to the apparent loss of the enterotoxin P gene (*sep*) noted by Challagundla et al. (*8*), isolates from this clade uniformly lacked the *splD* gene encoding serine protease D (Figure 1).

### Monocytic Evasion

**Figure 4:**
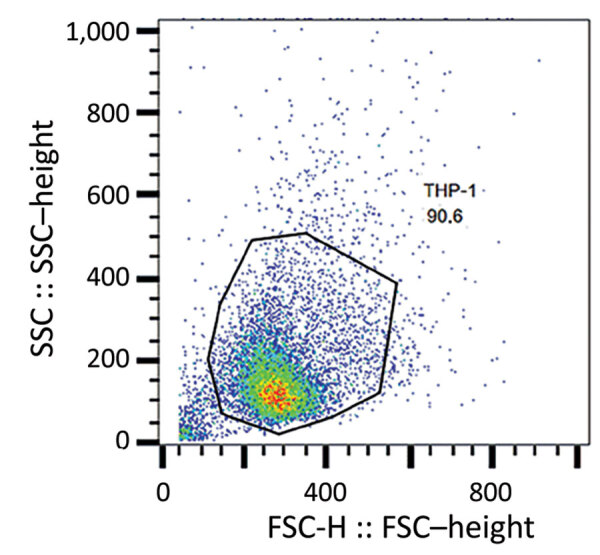
Scatter plot representing gating strategy for identifying monocytes in the FSC-H versus SSC-H analysis of methicillin-resistant *Staphylococcus aureus* isolates, Rio de Janeiro, Brazil, 2014–2017. Representative flow cytometry chart shows the acquisition of THP-1 cells not exposed to methicillin-resistant *Staphylococcus aureus*. FSC-H, forward scatter height; SSC-H, side scatter height.

**Figure 5:**
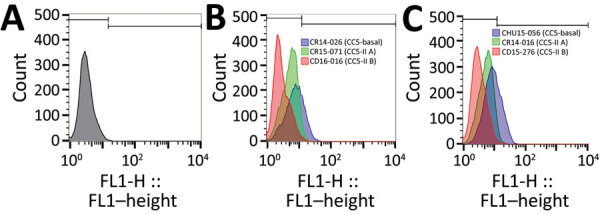
Histograms showing count versus green fluorescence intensity of THP-1 cells exposed or not to MRSA isolates, Rio de Janeiro, Brazil, 2014–2017. A) Acquisition of THP-1 cells not exposed to MRSA. B) Acquisition of THP-1 cells exposed to representative strains of 3 MRSA lineages. Blue indicates ST5-SCC*mec*IV-t002, strain CR14-026 (CC5-Basal lineage); green indicates ST5-SCC*mec*II-t539, strain CR15-071 (CC5-IIA lineage); and red indicates ST105-SCC*mec*II-t002, strain CD16–016 (CC5-IIB lineage). C) Acquisition of THP-1 cells exposed to representative strains of 3 MRSA lineages. Blue indicates ST5-SCC*mec*IV-t002, strain CHU15–056 (CC5-Basal lineage); green indicates ST5-SCC*mec*II-t539, strain CR14–016 (CC5-IIA lineage); and red indicates ST105-SCC*mec*II-t002 strain CD15–276 (CC5-IIB lineage). CC, clonal complex; FL1-H, forward light 1 height; MRSA, methicillin-resistant *Staphylococcus aureus*; SCC, staphylococcal cassette chromosome; ST, sequence type.

**Figure 6:**
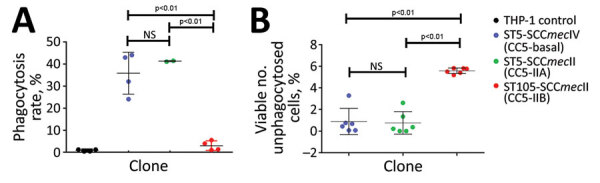
Scatter plots showing evasion of phagocytosis by MRSA isolates, Rio de Janeiro, Brazil, 2014–2017. A) Phagocytosis rates for representatives of the phylogenetic groups CC5-Basal, CC5-IIA, and CC5-IIB. Four independent experiments were conducted for each lineage using 1 fluorescence-activated single cell sorting determination for each experiment. Horizontal lines indicate means; whiskers indicate SDs. B) Viable count of unphagocytosed (free) bacteria after 30-min interaction with THP-1 monocytes. Six independent experiments were considered, with 2 replicates per lineage. Horizontal lines indicate means; whiskers indicate SDs. Statistical analyses conducted using 1-way analysis of variance and Tukey multiple comparison test. CC, clonal complex; NS, not significant; MRSA, methicillin-resistant *Staphylococcus aureus*; SCC, staphylococcal cassette chromosome; ST, sequence type.

To better ascertain differences in pathogenicity of RdJ isolates, we assessed the in vitro phagocytosis rate and viable counts of unphagocytosed (free) bacteria (Figures 4,5,6). Representative RdJ isolates showed very low rates of phagocytosis/host cell association (2.9%) compared with representatives of other CC5 lineages: 41.3% for ST5(CC5)-SCC*mec*II-t539 and 35.8% for ST5(CC5)-SCC*mec*IV-t002 strains (Figure 6, panel A). In addition, after a 30-minute interaction with THP-1 monocytes, the RdJ strains showed higher survival rates (5.58%) than other lineages: 0.88% for ST5(CC5)-SCC*mec*IV-t002 and 0.76% for ST5(CC5)-SCC*mec*II-t539 (Figure 6, panel B).

## Discussion

Using molecular typing and phylogenetic analysis, we identified a third epidemic lineage of MRSA in Rio de Janeiro. CC5, and to a lesser extent CC30, have become the most prevalent MRSA lineages in Rio de Janeiro hospitals, replacing the previously dominant ST1(CC1)-SCC*mec*IV lineage, which had replaced the BEC lineage ST239(CC8)-SCC*mec*III during 2004–2008 (*4*). At the time when the ST1(CC1)-SCC*mec*IV lineage replaced BEC, CC5 comprised only 10% of isolates (*4*); CC5 now constitutes >60% of isolates. Previously dominant clones, especially BEC, carried resistance to many non–β-lactam antimicrobial drugs, antiseptics, and heavy metals whereas the currently dominant strains are more susceptible (*1*).

Although CC5-SCC*mec*II was the predominant genotype in our sample, the proportions of the second and third most frequent genotypes, CC5-SCC*mec*IV and *lukSF-PV*–positive CC30-SCC*mec*IV, also had increased from prior studies (*4*). CC5-SCC*mec*IV (related to USA800), which was first isolated in children at a hospital in Portugal in 1992 (*26*), was overrepresented among patients <5 years of age in our sample. Some studies have suggested that this strain is more common among children (*27*), although the nature of this association remains unclear. The *lukSF-PV*–positive CC30-SCC*mec*IV genotype is related to the USA1100/Oceania South West Pacific clone (*1*), and is a distant relative of the historically epidemic and especially virulent phage type 80/81 lineage (*28*). We previously showed that, in contrast to the 80/81 lineage, ST30(CC30)-SCC*mec*IV MRSA from Brazil displays a natural attenuation of the Agr and SaeRS virulence regulators (*29*), which might explain why this lineage was responsible for only 9.6% of BSIs in this study.

The large number of MRSA isolates genotyped in this study enabled us to assess the distribution of MRSA genotypes by patient age and sites of infection or colonization. We identified associations between the CC5-SCC*mec*II genotype, BSIs, and older age, possibly because of the increased virulence or invasiveness of this genotype. The CC5-SCC*mec*II genotype also is found in the USA100 lineage ST5(CC5)-SCC*mec*II that was dominant among hospitals in the United States during the late 1990s (*30*), before the emergence of the USA300 clone (*31*). USA100 is still found in hospitals in the United States (*32*) and around the world (*1*).

In our sample, most (75/114; 65.8%) CC5-SCC*mec*II isolates belonged to ST105 and shared *spa*-type t002, suggesting the emergence of a new clone. ST105(CC5)-SCC*mec*II strains have previously infected humans and domestic animals (*33*), and 4 isolates from this lineage were reported in a hospital in São Paulo (*7*). Reports from other countries have occasionally shown a substantial prevalence of this lineage, including a study that showed colonization among 22.4% of patients admitted to a hospital in Pennsylvania, USA (*34*). Another study showed that ST105(CC5)-SCC*mec*II was the predominant lineage among patients who had MRSA BSI in Switzerland (*35*). In Portugal, ST105(CC5)-SCC*mec*II has been reported as the most abundant MRSA colonizing patients >60 years of age (*33*); a multicenter study identified this lineage as the second most common clone among patients who had BSIs (*36*). The first vancomycin-resistant *S. aureus* isolate in Portugal belonged to this lineage (*37*), a troubling finding because most vancomycin-resistant *S. aureus* isolates have belonged to the CC5 lineage (*25*).

Few studies exist on the molecular epidemiology of MRSA in Brazil and in other countries from South America; existing studies are based on a limited number of samples (*5*–*7*,*9*). As a result, the full extent of the dissemination of the ST105-SCC*mec*II-t002 genotype in Latin America is unknown. Since the late 2000s, ST105-SCC*mec*II-t002 has been reported as the second or third most frequent MRSA lineage in hospitals in the United States and some countries in Europe (*33*–*35*). For example, researchers documented an outbreak of ST105-SCC*mec*II-t002 MRSA among 18 neonates at Mount Sinai Hospital (New York, NY, USA) during 2014–15 (*38*). In addition, ST105 isolates comprised 87.5% of delafloxacin-resistant MRSA strains collected in 7 hospitals in New York (*39*). Altogether, these data show that ST105 is a major MRSA lineage not only in Rio de Janeiro but also in other countries. ST105-SCC*mec*II-t002 also might have spread in other regions of Brazil; therefore, more studies are needed to better track and investigate this lineage.

We used Bayesian molecular clock analysis to estimate the expansion of the RdJ clade in Rio de Janeiro in 2009 (95% highest posterior density 2007–2010), which is consistent with previous estimates that date the origin of the ST105 lineage to the mid-1990s (*8*). The ST105 clade is characterized by a lack of virulence genes that are common among other CC5 strains. All ST105(CC5)-SCC*mec*II isolates lacked the *sep* gene encoding enterotoxin P, as noted by Challagundla et al. (*8*). In addition to its emetic properties, enterotoxin P is a superantigen that induces T-cell proliferation and production of proinflammatory cytokines (*40*). ST105(CC5)-SCC*mec*II-t002 strains showed resistance to fluoroquinolones, macrolides, and lincosamides. ST105(CC5)-SCC*mec*II isolates also lacked the serine protease encoding gene *splD*, despite the presence of the *splABCF* genes of the *spl* operon. Although the specific role of SplD in *S. aureus* pathogenesis is not known, some researchers have proposed that Spl serine proteases might use proteolysis to modulate host proteins critical to bacterial pathogenesis (*41*). Future work should address implications of the absence of SplD in the ST105(CC5)-SCC*mec*II lineage.

Compared with representatives of the ST5-SCC*mec*II-t539 and ST5-SCC*mec*IV-t002 lineages, representative isolates of the RdJ clade showed increased evasion of phagocytosis mechanisms upon exposure to monocytic cells (i.e., THP-1). Multiple factors, including phagocytosis rate and the activity of toxic compounds released by monocytes, might affect the number of viable unphagocytosed bacterial cells (*42*). Moreover, we observed an increased number of viable RdJ free cells. The basis of this phenotype is unclear and deserves further study. Le Pabic et al. (*43*) implicated the small noncoding RNA, SprC, and its effect on regulation of the major autolysin Atl in *S. aureus* evasion of phagocytosis by human monocytes and macrophages. However, we did not find any differences in the *sprC* gene of the 6 representative strains tested, suggesting that the observed evasion might be multifactorial, probably linked to the production of several bacterial molecules (*42*).

One limitation of this study is the lack of more extensive clinical data such as the presence of indwelling catheters or lines and underlying conditions that might have affected our estimates. The association between ST105-SCC*mec*II-t002 and BSIs was attenuated when accounting for other variables such as hospital type and year of isolation, but still might be of clinical relevance. Access to more extensive clinical data would enable further exploration of this relationship. In addition, our reliance on samples from Rio de Janeiro might have affected our phylogenetic analysis; focused sampling in other geographic locations might show a more widespread epidemic.

In summary, we uncovered a new MRSA clone in hospitals in the Rio de Janeiro metropolitan area. Our findings emphasize the dynamic nature of the local rise and decline of various MRSA clones. In addition, these data indicate that MRSA clonal dynamics also might be associated with different manifestations of disease and host factors, such as age. This analysis revealed the emergence of a novel multidrug-resistant MRSA clone associated with BSIs. This association might be critical for assessing the clinical and epidemiologic risks associated with the spread of this clone and the biologic basis for its putative enhanced invasiveness.

Appendix 1 Additional data on multidrug-resistant methicillin-resistant *Staphylococcus aureus* associated with bacteremia and monocyte evasion, Rio de Janeiro, Brazil.

Appendix 2 Additional data on sequenced isolates of multidrug-resistant methicillin-resistant *Staphylococcus aureus* associated with bacteremia and monocyte evasion, Rio de Janeiro, Brazil.

Appendix 3 Additional methods used to detect multidrug-resistant methicillin-resistant *Staphylococcus aureus* associated with bacteremia and monocyte evasion, Rio de Janeiro, Brazil.
