# Supplementary material for: Multidrug-Resistant Methicillin-Resistant Staphylococcus aureus Associated with Bacteremia and Monocyte Evasion, Rio de Janeiro, Brazil
Source: Emerg Infect Dis. 2021 Nov;27(11):2825–35. doi: 10.3201/eid2711.210097 (PMC8544994; doi:10.3201/eid2711.210097)
Supplement: Appendix 3 — Additional methods used to detect multidrug-resistant methicillin-resistant Staphylococcus aureus associated with bacteremia and monocyte evasion, Rio de Janeiro, Brazil. [file 21-0097-Techapp-s3.pdf]

Article DOI: https://doi.org/10.3201/eid2711.210097

Multidrug-Resistant Methicillin-Resistant *Staphylococcus aureus* Associated with Bacteremia and Monocyte Evasion, Rio de Janeiro, Brazil

Appendix 3

Appendix Methods

Phylogenetic Analysis and Divergence Times

A maximum-likelihood tree was constructed for 661 genomes; 179 clonal complex 5 (CC5) genomes from our collection and 482 CC5 assembled genomes available from GenBank (*1*), chosen from the list provided by Challagundla et al. (*2*). Read mapping to the N315 reference genome (GenBank accession no. BA000018.3) and variant calling were performed by using Snippy version 4.6.0 (https://github.com/tseemann/snippy). A single-nucleotide polymorphism (SNP) alignment produced by Snippy was used to infer an initial phylogenetic tree in RAxML version 8.2.4 (*3*) by using an ascertainment bias correction and general time-reversible substitution model (*4*) accounting for among-site rate heterogeneity using the gamma distribution and 4 rate categories (ASC_GTRGAMMA model) (*5*) for 100 individual searches with maximum parsimony random-addition starting trees. Node support was evaluated with 100 nonparametric bootstrap pseudoreplicates (*6*). For better visualization, the tree was edited by using Interactive Tree of Life version 4.2.3 (*7*).

To estimate the emergence time of the sequence type 105 isolates from Rio de Janeiro (RdJ clone), we used a Bayesian phylogenetic framework implemented in BEAST version 2.6.0 (*8*). For this analysis, genomes that failed our Mash Screen (*9*) cutoffs (i.e., a genome has a hit that is not *S. aureus* and has Mash identity of >0.85 and shared-hashes value of >100) were excluded, leading to a total of 73/82 genomes included. To select the closest complete reference genome from GenBank that would capture all genomic regions in the 73 RdJ strains, we annotated genomes in Prokka 1.14.5 (*10*) by using WhatsGNU topgenome (-t) option (*11*).This

approach identified FCFHV36 (GenBank accession no. CP011147.1) as the GenBank hit that was the closest to all 73 genomes and shared an average of 2,485 protein open reading frames with each genome. Read mapping to the complete reference genome FCFHV36 and variant calling were performed by using Snippy. A SNP alignment produced by Snippy was used to infer an initial phylogenetic tree in RAxML version 8.2.4 (*3*) by using an ascertainment bias correction and general time-reversible substitution model (*4*) to account for among-site rate heterogeneity using the gamma distribution and 4 rate categories (ASC_GTRGAMMA model) (*5*) for 100 individual searches with maximum parsimony random-addition starting trees. Node support was evaluated with 100 nonparametric bootstrap pseudoreplicates (*6*). The initial ML newick tree and the whole-genome alignment, including SNPs and invariant sites, were used as inputs for ClonalFrameML (*12*) to infer recombination using 100 pseudo-bootstrap replicates. A SNP alignment was then called from the resultant alignment using the SNP-sites tool (*13*).

The SNP alignment was then used to estimate divergence times in BEAST (*8*). To gauge the potential for temporal signal of our dataset, we used a regression of root-to-tip genetic distance versus isolation time as a diagnostic tool, as implemented in TempEst version 1.5.3 (*14*). A positive correlation between genetic divergence and isolation time was observed ($R^2 = 0.4177$), indicating suitability of this dataset for downstream molecular clock analysis in BEAST. The Hasegawa–Kishino–Yano nucleotide substitution model was used (*15*) with estimated base frequencies, and ascertainment bias for variable-only sites was corrected by editing the XML file and factoring in the number of invariable sites based on fully sequenced genomes (https://www.beast2.org/2019/07/18/ascertainment-correction.html). We implemented a strict clock model with a random starting tree, a coalescent constant population (*16*), and a uniform prior probability distribution of 10–3–10–7 substitutions/site/year. The chain length was set at 100 million Markov chain Monte Carlo steps with a 10,000-step thinning and was run 3 independent times. We also implemented an uncorrelated lognormal relaxed clock model in BEAST. The chain length was set at 200 million Markov chain Monte Carlo steps with a 20,000-step thinning. Since clock rate heterogeneity among branches was not large in the relaxed clock model runs (i.e., the 95% highest posterior density interval for the ucld.Stdev and rate.coefficientOfVariation parameters were 0–0.27), and the trees and divergence times produced by the strict (Figure 3) and relaxed clock techniques were largely indistinguishable. Sampling from the prior without sequence data also was used to assess whether the data are

informative on different parameters by looking for a departure of the posterior distribution from the prior. Three independent strict clock runs were combined post convergence using LogCombiner version 2.4.7 and resample posterior distribution of the tree files at a lower frequency (30,000 steps), following inspections of the sampled parameters and their effective sample size (i.e., >728) in Tracer version 1.7.1. The chronograms were plotted on the basis of the maximum clade credibility tree using the TreeAnnotator program from the BEAST package after removing 10% of the first posterior samples as a burn-in and were visualized in FigTree version 1.4.3. The tree file for Bayesian analysis RAxML is available in Mendeley data (https://data.mendeley.com/datasets/3pz36bdb63/1).

References

1. Sayers EW, Cavanaugh M, Clark K, Ostell J, Pruitt KD, Karsch-Mizrachi I. GenBank. Nucleic Acids Res. 2019;47:D94–9. PubMed https://doi.org/10.1093/nar/gky989

2. Challagundla L, Reyes J, Rafiqullah I, Sordelli DO, Echaniz-Aviles G, Velazquez-Meza ME, et al. Phylogenomic classification and the evolution of clonal complex 5 methicillin-resistant *Staphylococcus aureus* in the Western Hemisphere. Front Microbiol. 2018;9:1901. PubMed https://doi.org/10.3389/fmicb.2018.01901

3. Stamatakis A. RAxML version 8: a tool for phylogenetic analysis and post-analysis of large phylogenies. Bioinformatics. 2014;30:1312–3. PubMed https://doi.org/10.1093/bioinformatics/btu033

4. Lanave C, Preparata G, Sacone C, Serio G. A new method for calculating evolutionary substitution rates. J Mol Evol. 1984;20:86–93. PubMed https://doi.org/10.1007/BF02101990

5. Yang Z. A space-time process model for the evolution of DNA sequences. Genetics. 1995;139:993–1005. PubMed https://doi.org/10.1093/genetics/139.2.993

6. Felsenstein J. Confidence limits on phylogenies: an approach using the bootstrap. Evolution. 1985;39:783–91. PubMed https://doi.org/10.1111/j.1558-5646.1985.tb00420.x

7. Letunic I, Bork P. Interactive tree of life (iTOL) v3: an online tool for the display and annotation of phylogenetic and other trees. Nucleic Acids Res. 2016;44:W242–5. PubMed https://doi.org/10.1093/nar/gkw290

8. Bouckaert R, Vaughan TG, Barido-Sottani J, Duchêne S, Fourment M, Gavryushkina A, et al. BEAST 2.5: An advanced software platform for Bayesian evolutionary analysis. PLOS Comput Biol. 2019;15:e1006650. PubMed https://doi.org/10.1371/journal.pcbi.1006650

9. Ondov BD, Starrett GJ, Sappington A, Kostic A, Koren S, Buck CB, et al. Mash Screen: high-throughput sequence containment estimation for genome discovery. Genome Biol. 2019;20:232. PubMed https://doi.org/10.1186/s13059-019-1841-x

10. Seemann T. Prokka: rapid prokaryotic genome annotation. Bioinformatics. 2014;30:2068–9. PubMed https://doi.org/10.1093/bioinformatics/btu153

11. Moustafa AM, Planet PJ. WhatsGNU: a tool for identifying proteomic novelty. Genome Biol. 2020;21:58. PubMed https://doi.org/10.1186/s13059-020-01965-w

12. Didelot X, Wilson DJ. ClonalFrameML: efficient inference of recombination in whole bacterial genomes. PLOS Comput Biol. 2015;11:e1004041. PubMed https://doi.org/10.1371/journal.pcbi.1004041

13. Page AJ, Taylor B, Delaney AJ, Soares J, Seemann T, Keane JA, et al. *SNP-sites*: rapid efficient extraction of SNPs from multi-FASTA alignments. Microb Genom. 2016;2:e000056. PubMed https://doi.org/10.1099/mgen.0.000056

14. Rambaut A, Lam TT, Max Carvalho L, Pybus OG. Exploring the temporal structure of heterochronous sequences using TempEst (formerly Path-O-Gen). Virus Evol. 2016;2:vew007. PubMed https://doi.org/10.1093/ve/vew007

15. Hasegawa M, Kishino H, Yano T. Dating of the human-ape splitting by a molecular clock of mitochondrial DNA. J Mol Evol. 1985;22:160–74. PubMed https://doi.org/10.1007/BF02101694

16. Griffiths RC, Tavaré S. Sampling theory for neutral alleles in a varying environment. Philos Trans R Soc Lond B Biol Sci. 1994;344:403–10. PubMed https://doi.org/10.1098/rstb.1994.0079

Appendix 3 Table 1. Phylogenetic groups of the methicillin-resistant *Staphylococcus aureus* CC5 strains randomly selected for phagocytosis and enumeration of viable unphagocytosed (free) bacterial cells, Rio de Janeiro, Brazil, 2014–2017*

Strain*	Lineage	Clade position†
CR14–026	ST5(CC5)-SCC*mec*IV-t002	CC5-Basal
CHU15–056	ST5(CC5)-SCC*mec*IV-t002	CC5-Basal
CR14–016	ST5(CC5)-SCC*mec*II-t539	CC5-IIA
CR15–071	ST5(CC5)-SCC*mec*II-t539	CC5-IIA
CD15–276	ST105(CC5)-SCC*mec*II-t002	CC5-IIB
CD16–016	ST105(CC5)-SCC*mec*II-t002	CC5-IIB

*Two MRSA strains from Rio de Janeiro were selected by lottery from each of the 3 clades (i.e., CC5-Basal, CC5-IIA, and CC5-IIB) to which the CC5 MRSA were allocated. CC5, clonal complex 5.
†Clades were named according to the classification of Challagundla et al. (*2*).

Appendix 3 Table 2. Molecular characterization of 29 methicillin-resistant *Staphylococcus aureus* non–clonal complex 5 strains, Rio de Janeiro, Brazil, 2014–2017*

Multilocus sequence type	SCC*mec* type	Related clone	*lukSF*	No. isolates
ST30(CC30)	IV	USA1100/OSPC	+	12
ST1(CC1)	IV	USA400/MW2	–	7
ST8(CC8)	IV	USA300	+	3
ST239(CC8)	III	BEC	ND	3
ST45(CC45)	II	USA600	ND	2
ST3603(CC45)	IV	USA600	ND	1
ST188(CC188)	IV	Sporadic (rare) MRSA	ND	1

**lukSF*, genes encoding Panton-Valentine leukocidin; ND, not done; ST, sequence type; +, positive; –, negative.
